# New Performance Measurement Framework for Realizing Patient-Centered Clinical Decision Support: Qualitative Development Study

**DOI:** 10.2196/68674

**Published:** 2025-04-30

**Authors:** Prashila Dullabh, Courtney Zott, Nicole Gauthreaux, James Swiger, Edwin Lomotan, Dean F Sittig

**Affiliations:** 1 Health Sciences Department NORC at the University of Chicago Washington, DC United States; 2 Center for Evidence and Practice Improvement Agency for Healthcare Research and Quality Rockville, MD United States; 3 Informatics Review, LLC Lake Oswego, OR United States

**Keywords:** patient-centered, measurement, health care, clinical decision support, artificial intelligence, AI

## Abstract

**Background:**

Patient-centered clinical decision support (PC CDS) exists on a continuum that reflects the degree to which its knowledge base, data, delivery, and use focus on patient needs and experiences. A new focus on value-based, whole-person care has resulted in broader development of PC CDS technologies, yet there is limited information on how to measure their performance and effectiveness. To address these gaps, there is a need for more measurement guidance to assess PC CDS interventions.

**Objective:**

This paper presents a new framework that incorporates patient-centered principles into traditional health IT and clinical decision support (CDS) evaluation frameworks to create a unified guide to PC CDS performance measurement.

**Methods:**

We conducted a targeted literature review of 147 sources on health IT, CDS, and PC CDS measurement and evaluation to develop the framework. Sources were reviewed if they included the sociotechnical components relevant to PC CDS, covered the full IT life cycle of PC CDS, and addressed measurement considerations at different user and system levels. We then validated and refined the measurement framework through key informant interviews with 6 experts in measurement, CDS, and clinical informatics. Throughout the framework development, we gathered feedback from a 7-member expert committee on the methods, findings, and the framework’s relevance and application.

**Results:**

The PC CDS performance measurement framework includes 6 domains: safe, timely, effective, efficient, equitable, and patient centered. The 6 domains contain 34 subdomains that can be selected to assess performance, depending on the type of PC CDS intervention or the specific research focus. In addition, there are 4 levels of aggregation at which subdomains can be measured (individual, population, organization, or IT system) that account for the multilevel impact of PC CDS. We provide examples of measures and approaches to patient centeredness for each subdomain, followed by 2 illustrative use cases demonstrating the framework application.

**Conclusions:**

This framework can be used by researchers, health system leaders, informaticians, and patients to understand the full breadth of performance and impact of PC CDS technology. The framework is significant in that it (1) covers the entire PC CDS life cycle, (2) has a direct focus on the patient, (3) covers measurement at different levels, (4) encompasses 6 independent but related domains, and (5) requires additional research and development to fully characterize all domains and subdomains. As the field of PC CDS matures, researchers and evaluators can build upon the framework to assess which components of PC CDS technologies work; whether PC CDS technologies are being used as anticipated; and whether the intended outcomes of delivering evidence-based, patient-centered care are being achieved.

## Introduction

### Background

In response to a growing body of research on suboptimal health care quality in the United States [1,2], organizations delivering health care are amid an unprecedented transition in how they approach patient care and reimbursement. The new focus is on value-based, whole-person care that prioritizes patient values, goals, needs, and preferences to achieve positive health outcomes. This new approach to care calls for health care organizations to become “learning health systems” to generate and integrate knowledge for continuous improvement and innovation [3], which includes advancement of health IT and promotion of personalized medicine, patient involvement in the clinical decision-making process, and treatment recommendations guided by evidence-based guidelines.

Clinical decision support (CDS) technology (eg, electronic health record [EHR] prompts and alerts and diagnostic and treatment guidance for clinicians based on clinical guidelines) aims to achieve these priorities but has traditionally focused on supporting clinicians at the point of care. Technology that supports patients and incorporates patient-centered knowledge and patient-generated health data, known as patient-centered CDS (PC CDS), holds promise for advancing value-based, whole-person care [4,5]. PC CDS encompasses a spectrum of decision-making tools that significantly incorporate patient-centered factors related to knowledge, data, delivery, and use. Knowledge refers to the use of comparative effectiveness research or patient-centered outcomes research findings. Data refer to the incorporation of patient-generated health data, patient preferences, social determinants of health (SDOH), and other patient-specific information. Use refers to directly engaging patients and caregivers across different settings. Finally, delivery focuses on facilitating bidirectional information exchange in support of patient-centered care, including shared decision-making [6].

CDS developers commonly use the 5 rights [7] as a benchmark for what makes a successful intervention, and several recent assessments of the CDS field have been published [8-10]. However, less is known about the emerging field of PC CDS [6]. While PC CDS has the potential to improve patient outcomes and enhance quality of care, it also has the potential to contribute to clinician burnout and undermine patient safety if it provides incorrect, irrelevant, uninformative, or nonspecific alerts and recommendations [11]. Measuring user acceptance performance is critical to understanding how PC CDS technology can achieve the 5 rights and prioritize outputs that are meaningful to patients and clinicians, as well as what unintended consequences may exist. PC CDS performance measurement is a complex undertaking given the intricacies of interventions, variety of data sources (eg, EHRs, remote devices, and apps), and nascency of the field. Without the understanding of what leads to a successful PC CDS intervention, PC CDS technology development, use, effectiveness, and scalability will remain limited. To ensure PC CDS technology is safely and effectively integrated into health care delivery systems, further research and measurement are needed to adapt to the goals of a patient-centered approach and facilitate understanding of the factors that lead to successful implementation.

### Objectives

This paper presents a new framework that incorporates patient-centered principles into traditional health IT and CDS evaluation frameworks to create a unified guide to PC CDS performance measurement.

## Methods

We used three methods in developing the new performance measurement framework: (1) literature review, (2) key informant interviews, and (3) feedback from an expert committee (refer to the Acknowledgments section for expert committee members).

### Literature Review

Our literature review aimed to identify existing measurement frameworks and types of measurements used to assess health IT, CDS, and PC CDS technology performance. We began with a snowball approach, using a starting set of 10 peer-reviewed health IT and CDS measurement frameworks and measurement-related publications compiled by 1 of the authors (DFS) with expertise in CDS measurement and evaluation. We conducted backward citation tracking of the starting set of papers and then conducted a title and abstract screening to determine whether each article met the eligibility criteria (Textbox 1).

The team reviewed the full text of each source and extracted information according to three predetermined data abstraction domains: (1) whether the source was a formal evaluation or measurement framework; (2) whether it was validated; and (3) whether the source’s findings specified levels of measurement (eg, organization and individual), measure domains, measure subdomains, and specific measures. Articles that did not meet ≥1 of these criteria were excluded from the review. This resulted in 44 peer-reviewed publications.

**Eligibility criteria for literature review.**

**Eligibility criteria**

- Covered at least 1 of the following 3 areas: first, the article included sociotechnical components relevant to patient-centered clinical decision support (PC CDS), such as human, organizational, environmental, and technical factors. Inclusion of these factors is important, as PC CDS needs to operate in complex health care systems, and the interaction of many different factors (including technology, people, environment, workflow, and organizational factors) has implications for PC CDS adoption and use [12]. Second, the article discussed the full IT life cycle of PC CDS design, development, implementation, use, and measurement. Comprehensive evaluation strategies need to account for collaboration across both technical and clinical disciplines through the full life cycle of system development and use [13]. Third, the article considered measurements at different levels (eg, individual, system, and health IT). PC CDS measurements need to occur at different levels to fully understand the factors that impede adoption and use at both user and system levels.
- Published as a peer-reviewed research article (ie, not abstracts, viewpoints, commentaries, editorials, or protocols).
- Full text available in English.

To begin constructing the domains and subdomains of the framework from these sources, we first used an inductive approach where 2 researchers mapped similar domains and subdomains within the literature to identify the most mentioned concepts. Two senior members of the research team (PD and DFS) then reviewed the mapping, discussed disagreements, and agreed on modifications to arrive at final domains and associated subdomains. After discovering that many of the domains and subdomains in the literature focused on aspects of quality measurement, we conducted another targeted literature review to identify existing health care quality frameworks that covered our identified domains. As a result of this review, we selected the National Academy of Medicine’s (NAM’s) 6 domains of health care quality [14] as the organizing construct for our framework because it included all our identified domains, and it is well known in the CDS field.

To define the framework’s 34 subdomains, the team extracted definitions from the full text of each source. In cases where definitions or descriptions were not explicitly stated, we conducted supplemental searches specific to each subdomain in PubMed and Google Scholar to find more information. We also conducted supplemental searches to identify specific examples of measures in the subdomain and to describe the relationship of each domain and subdomain with PC CDS interventions. Given that PC CDS is a relatively new term, we used search terms encompassing key areas of CDS, such as “digital health” and “shared decision-making,” in combination with the relevant measurement concept (Textbox 2).

**Figure 1:**
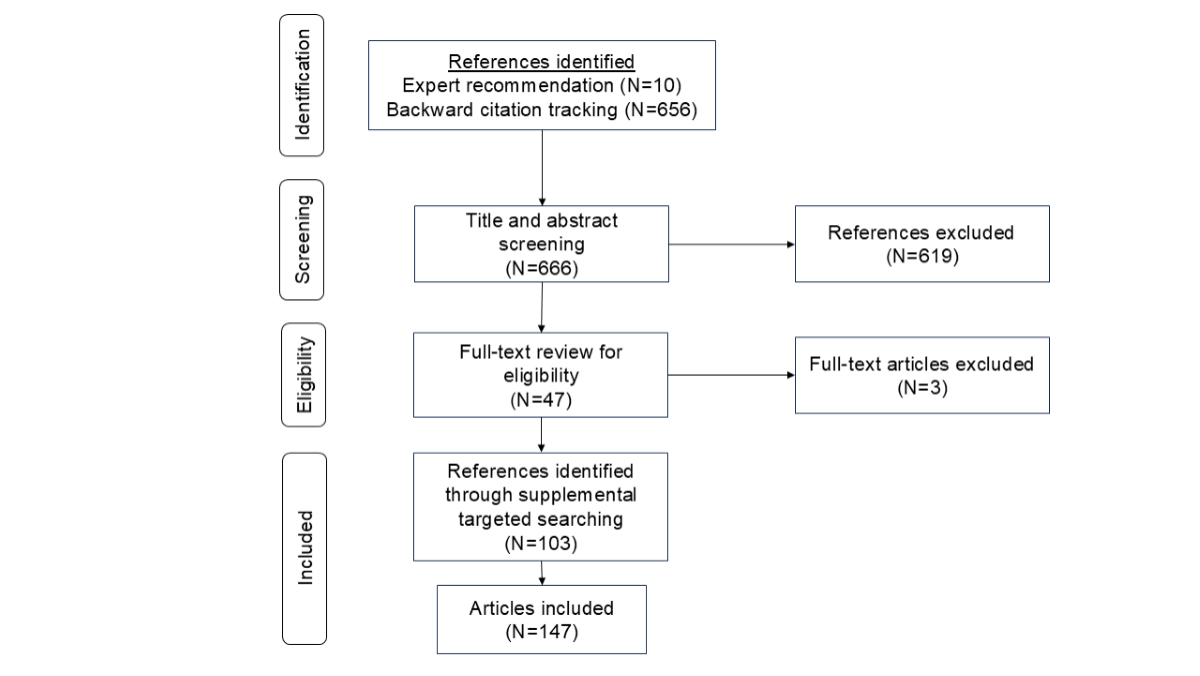
Flow diagram for literature review.

We screened titles and abstracts and excluded publications that were not peer-reviewed research articles, were not available in English, did not involve a PC CDS technology, or did not measure the appropriate measure domain. This yielded an additional 103 peer-reviewed articles (approximately 3 papers per each of the 34 subdomains). The final search included 147 sources (Figure 1). To manage the screening process for all searches, we used Zotero (version 6.0; Corporation for Digital Scholarship) for reference management and Microsoft Excel for inclusion and exclusion decisions.

**Example search terms.**

**Keyword concepts and keywords**

- Patient-centered clinical decision support: (“patient-centered clinical decision support” OR “PC CDS” OR “clinical decision support” OR “CDS”)
- Measurement: AND (“measure” OR “metrics” OR “evaluation” OR “assessment” OR “performance measure”)
- Measure domain or subdomain of interest: AND “[domain of interest]”; OR “[subdomain of interest]”

### Key Informant Interviews

Using the network of the Clinical Decision Support Innovation Collaborative [15], we identified a convenience sample of 8 potential informants to ensure informants represented different PC CDS stakeholder groups (patient representatives, clinicians, informaticians, measurement experts, and CDS developers). Specifically, the research team identified an initial list of known experts in the field of CDS and measurement. We then used a snowball approach to receive referrals from key informants on other experts to interview. Ultimately, we recruited 6 key informants—2 clinicians, 2 informaticians, and 2 measurement experts—to seek feedback on the framework and validate the measurement domains and subdomains.

Informants received the draft framework with the 6 NAM domains in advance of the interviews (Multimedia Appendix 1 [14]). During the 60-minute interviews, we asked informants to provide feedback in three key areas: (1) perspectives on how we organized the domains and subdomains, (2) any gaps in the domains and subdomains, and (3) any refinements to the existing domains and subdomains. Transcript-style notes were created after each interview. We analyzed information from the interviews using qualitative thematic synthesis [16], using an inductive approach with simultaneous phases of data collection and analysis. After each interview, 2 senior research team members (PD and DFS), who developed the draft framework and have expertise in qualitative coding, reviewed the transcript-style notes. They refined the themes identified in each interview and mapped them to the domains and subdomains identified in the literature review. Coding was conducted by 2 research analysts using Microsoft Excel. Analysts coded interviewee feedback into 4 overarching areas: subdomains to add, remove, recategorize, or refine. The research team discussed the findings within each code through biweekly collaborative sessions, where they mapped results to domains identified in the literature and resolved discrepancies through consensus. To mitigate bias, the preliminary findings were also reviewed by a multidisciplinary 7-member expert panel to further enhance the framework development.

Key informant findings helped to refine themes in the following ways: (1) validating the organization of the domains and subdomains, (2) identifying gaps in the patient centered subdomain regarding decisional quality and knowledge acquisition, and (3) providing valuable feedback on the framing of health literacy and digital health literacy concepts.

### Expert Committee

The research team oversees a 7-member multidisciplinary expert committee that includes patient representatives, clinicians, informaticians, PC CDS developers, and payers. Committee members come from different academic, federal, and private institutions across the United States. Committee members were identified during the formation of the research collaborative to engage national leaders in advancing patient-centered technologies. To minimize bias that might serve the goals of 1 stakeholder group, the members were chosen to represent interdisciplinary professional backgrounds and perspectives.

The committee met on a quarterly basis through a web-based conferencing platform and provided feedback at 2 time points during the development of the framework: first, during the planning phases for the framework development, and second, during the analysis phase of findings from the literature review and interviews. Feedback from the committee was gathered via two 90-minute meetings. Meeting notes were summarized and analyzed for key themes. The expert committee validated the idea and need for a unified measurement framework for PC CDS performance, provided input on the organizing structure (NAM) and measurement domains for the framework, and reviewed the final framework.

### Ethical Considerations

The NORC at the University of Chicago Institutional Review Board (IRB00000967) determined that the protocol did not meet the definition of research with human participants and was therefore exempt from further review. As such, written informed consent was waived, yet interviewers obtained verbal consent from participants at the start of each interview after informing them that participation was voluntary. No identifiable information is included in this manuscript.

## Results

### Overview

We identified 6 measurement and evaluation frameworks and 2 systematic reviews of such frameworks (Textbox 3). These frameworks and systematic reviews cover general health IT as well as CDS-specific evaluation.

**Identified frameworks and systematic reviews.**

**Framework or systematic review and description**

- Human, Organization, and Technology-Fit Framework [17]: building on previous models of health information system (HIS) evaluation, this framework proposes 8 dimensions for evaluating HIS and numerous evaluation measures within those dimensions. The framework incorporates the concept of “fit” among human, organizational, and technical factors (ie, not only does performance matter on individual dimensions and measures, but these factors must be in alignment for HIS to be successful).
- Behavior and Acceptance Framework (BEAR) [18]: BEAR is an integrated conceptual framework that bridges the gap between behavioral change and technology acceptance aspects of clinical decision support (CDS) implementation. BEAR presents “constructs” that are “determinants of behavioral change and acceptance of CDS” and groups them into domains.
- Evaluation in Life Cycle of Information Technology [19]: this is an evaluation framework for electronic health record–integrated innovations to support evaluation activities at each of 4 IT life cycle phases: planning, development, implementation, and operation. The framework also proposes 3 levels at which evaluations can be conducted—society, user, and IT—and provides measurement exemplars within each level.
- Health IT Reference-Based Evaluation Framework [20]: this framework builds on a prior systematic review to propose 4 evaluation concepts comprising 12 evaluation components to assess health IT.
- Measures of success of computerized CDS systems: an overview of systematic reviews [21]: This overview is the first to focus on evaluation metrics of CDS, which were mapped according to the updated Information Systems Success Model by DeLone and McLean [22].
- Handbook of eHealth Evaluation: An Evidence-Based Approach [23]: this handbook provides a systematic overview of the different evaluation approaches to IT, with case examples that have been applied and reported for a wide range of health care systems and settings.
- Health information systems evaluation frameworks: a systematic review [24]: this paper analyzes studies on the evaluation of health information systems by applying a content, context, and process framework to address the “who,” “what,” “how,” “when,” and “why” of the evaluation processes used.
- Clinical, Human and Organizational, Educational, Administrative, Technical, Social framework [25]: This is an IT evaluation framework for health care that proposes six areas for assessment: (1) clinical, (2) human and organizational, (3) educational, (4) administrative, (5) technical, and (6) social. The framework recommends both qualitative and quantitative approaches to measuring the assessment areas.

Each framework offered several areas and concepts for measurement. Most began with an organizing construct for measurement and offered example measurement subcategories. However, the nature and specificity of the constructs and subcategories varied. Some frameworks were structured around broad ideas and concepts, while others began with a narrower focus. Due to the variation, we chose to categorize measurement concepts into 2 types: domains and subdomains. This enabled us to define domains as broad yet actionable measurement areas, within which more specific areas (subdomains) can be measured. We also found that the measure domains and subdomains included the structures, processes, and outcomes [26] associated with the PC CDS interventions, that is, measures related to the PC CDS technology and how it is performing, measures related to the impact of the PC CDS technology on patient and clinician behavior, and measures reflecting the impact of the PC CDS technology on clinical and other outcomes.

### Overview of the PC CDS Performance Measurement Framework

#### Overview

Our results begin with a high-level overview of the PC CDS performance measurement framework domains. We then provide further details about each domain and its subdomains, as well as illustrative measures. Given that PC CDS is a type of CDS, each domain and subdomain includes and builds on traditional CDS measures. To account for the unique features of PC CDS, we also specifically describe how to integrate patient-centered principles into each domain and subdomain to evaluate PC CDS performance.

**Figure 2:**
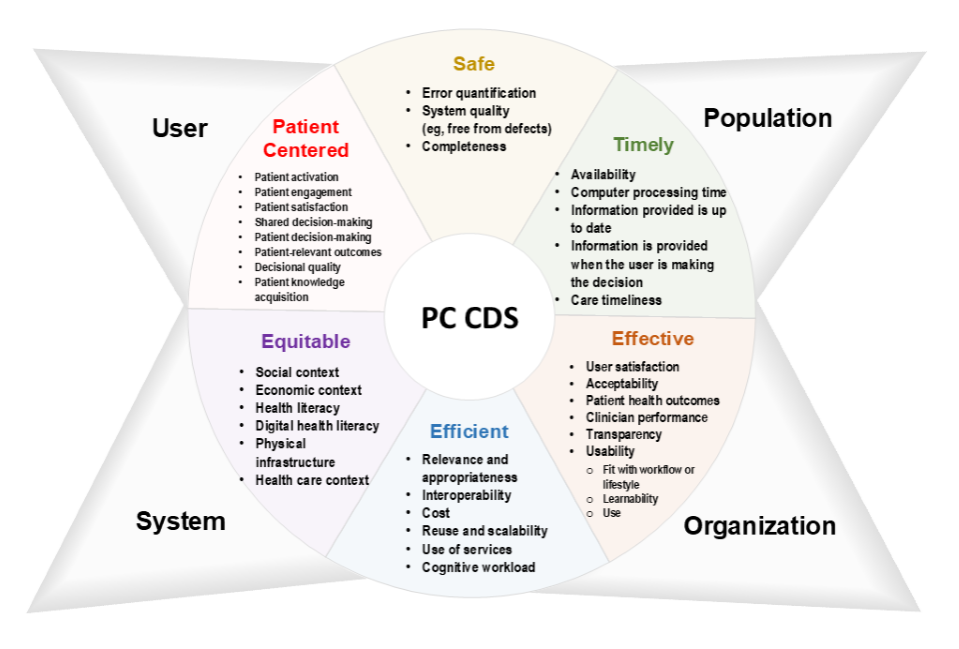
Patient-centered clinical decision support (PC CDS) performance measurement framework.

The complete measurement framework is shown in Figure 2. The framework accounts for the impact of the PC CDS intervention occurring at multiple levels (eg, individual user [patient or clinician], population of users, organization, and health IT system). The various levels at which the domains and subdomains can be measured are positioned around the framework, indicating that the measurement perspective may vary depending on the goals and scope of the intervention, as well as who is conducting the measurement and interested in the outcome.

The NAM’s six health care quality domains [14] are as follows:

1. Safe—avoiding harm to patients or users resulting from the system itself or the recommendations it provides.
2. Timely—reducing waits and sometimes harmful delays (eg, due to technology malfunction) for both those who receive and those who provide care.
3. Effective—providing functional, accessible systems and services based on scientific knowledge to all who could benefit and refrain from providing systems and services to those not likely to benefit (avoiding underuse and misuse, respectively).
4. Efficient—minimizing wasted or unnecessary effort by ensuring the system integrates smoothly into clinician and patient workflows, reduces user burden, and supports the intended user.
5. Equitable—providing interventions, advice, and care that account for SDOH and do not vary in quality because of personal characteristics.
6. Patient centered—providing interventions, advice, and care that are respectful of and responsive to individual patient preferences, needs, and values and ensuring patient values guide all clinical decisions.

#### Safe

PC CDS technology has the potential to improve patient safety by preventing adverse events. However, such technology can also cause adverse events (eg, when patient- or clinician-facing alerts are inappropriately overridden) if not designed, developed, or implemented correctly [27-33]. Concerns around patient safety and quality also prompted the US Food and Drug Administration to work with others in the federal government to develop a proposed strategy and recommendations for a risk-based framework for health information [34,35].

While not intended for legal compliance, the safety of PC CDS technology can be appropriately assessed by developers and implementers through measuring three subdomains: (1) error quantification, (2) system quality, and (3) completeness of data or other information (Table 1).

**Table 1:** Safe subdomain definitions.

Subdomain definition	Application to PC CDS^a^	Example measures
Error quantification: the degree to which a specific intervention leads to patient risks and safety-related reportable adverse events, including any unanticipated consequences to the patient, clinician, or health care organization [23,36].	Measures of errors should both (1) ensure patients are not experiencing negative clinical impacts (eg, adverse reaction to a drug due to an inappropriately overridden alert) [37], as well as (2) pay particular attention to symptoms patients may be experiencing [37].	Appropriateness of screening reminders Occurrence of severe or life-threatening patient safety events Patient-reported symptom burden or volatility Patient-reported adverse events
System quality: the degree to which the information and functions provided by the system meet the user’s needs or expectations and give user satisfaction; the degree to which the system is free from deficiencies or defects [18,38].	As the number of alerts sent directly to patients increases with PC CDS technology, measures of patient-centeredness should focus on ensuring the PC CDS can retrieve patient-contributed data accurately to drive alerts [39].	Knowledge accuracy, reliability, and validity Alert accuracy Algorithm accuracy Data retrieval accuracy Patient-reported issues with system functionality
Completeness: the degree to which the system provides all the information required by the user to make the intended decision or to perform the intended behavior [18,21,23,24,36,38,40].	Completeness should account for unique patient characteristics—patient-reported outcomes and patient preferences—that may impact care decisions. For example, when discussing treatment options for a patient on chemotherapy, the PC CDS technology needs to account for adverse patient-reported outcomes, such as peripheral neuropathy [41].	Degree of integration of relevant patient-contributed data into decision support artifact Degree to which relevant information is presented to the patient

^a^PC CDS: patient-centered clinical decision support.

#### Timely

PC CDS interventions aim to reduce delays for care teams and patients in providing or receiving care. When properly implemented, PC CDS interventions have been shown to enhance clinicians’ ability to deliver timely care [42] and help patients better manage their health. However, the CDS literature has documented numerous challenges to achieving this goal, including relevance of clinical recommendations, currency of clinical guidance, reliability of CDS tools, data availability, and more [43].

The timeliness of PC CDS interventions can be assessed by measuring five subdomains: (1) computer processing time, (2) whether information is up to date, (3) availability of the PC CDS technology, (4) whether information is provided when the user needs it, and (5) whether the patient receives care at the right time (Table 2). Measurements in each subdomain should be considered and be adapted to patient lifestyles, new patient-generated data types, different methods of delivery (eg, apps and patient portals), and patient preferences to form an adequate assessment of a PC CDS intervention’s impact on care timeliness. Patient preferences and experiences are critical to include in addition to standard measurements (eg, for care timeliness, a patient-centered measurement approach should examine both the time to care delivery and patient preferences around timing of treatment).

**Table 2:** Timely subdomain definitions.

Subdomain definition	Application to PC CDS^a^	Example measures
Computer processing time: the degree to which the time required for the computer to complete the work required to gather data, run the CDS^b^ algorithm, and generate an intervention is reduced [44].	Developers might be interested in how long it takes the PC CDS engine to gather data and run the algorithm necessary to process PGHD^c^ from remote devices and arrive at a recommendation.	Time for alert to fire Time for PC CDS artifact to incorporate relevant patient-contributed data
Information provided is up to date: the degree to which the information presented by the system is based on up-to-date input [18,21,36,40].	Use of PGHD, including PROs^d^, adds nuance to measurement of information timeliness (eg, a patient’s most recent blood glucose reading may not be the most relevant for long-term clinical decision-making).	Information resource includes the most current patient-provided information The artifact is updated with the latest evidence-based guidelines
Availability: the degree to which a system can be used when the user needs to use it [18,21,45,46].	Patient-centered evaluations should assess whether the patient encounters any technical issues when accessing the system [47,48].	Patient feedback on system glitches Patient-reported technical issues
Information is provided when the user is making the decision: the degree to which the necessary information presented by the PC CDS technology is available at the time it is needed [18,36,49].	Patient-centered evaluations should assess whether the PC CDS technology fires at the right time to support the patient in evidence-informed decision-making. Patient workflows or lifestyles are also key considerations for this subdomain.	Alert frequency (compared to patient’s expressed routines and preferences) Tool customizability (to enable patients to customize alerts to schedules)
Care timeliness: the degree to which the system can provide care in a timely manner after a need is recognized [21,50].	Measures should incorporate patients’ preferences regarding when an intervention should be delivered (eg, preferences to try medication or lifestyle changes first).	Time to care delivery Response time of the care team to patient inquiries submitted via PC CDS technology Response time of the care team to alerts

^a^PC CDS: patient-centered clinical decision support.

^b^CDS: clinical decision support.

^c^PGHD: patient-generated health data.

^d^PRO: patient-reported outcomes.

#### Effective

Effectiveness requires monitoring whether the PC CDS technology is benefiting those for whom it is intended. A landmark 2012 systematic review found that CDS interventions were generally effective in improving health care process measures, but evidence for effectiveness in areas such as improving clinical and economic outcomes was lacking [51]. Similarly, mixed findings on patient outcomes have been documented in more recent systematic CDS reviews in all health care settings [52]. Reviews of CDS effectiveness that focus on specific interventions or disease states (eg, medication-related CDS interventions) have also shown inconsistent results [53].

Another challenge is the prioritization of CDS recommendations based on their relevance and potential benefit to patients. Due to the influx of CDS alerts and reminders in EHR systems that interrupt workflows, it is common for clinicians to override alerts [54,55], with some studies showing that a third of CDS interventions have had no effect [13]. Given this, it is important to evaluate which outputs have the most value and perhaps not share low-level alerts with patients to reduce over alerting and enable more focused and collaborative decision-making [56].

Effectiveness of PC CDS interventions can be assessed by measuring six subdomains: (1) user satisfaction, (2) acceptability, (3) patient health outcomes, (4) usability, (5) clinician performance, and (6) transparency (Table 3).

**Table 3:** Effective subdomain definitions.

Subdomain definition	Application to PC CDS^a^	Example measures
User satisfaction: the degree to which a user is satisfied with their experience in using the system and with the system’s potential impact [21,23,24,36,57].	Evaluating different types of patients and clinicians as the end user ensures the PC CDS technology has the desired effects [36].	Willingness to recommend the PC CDS technology to others Satisfaction with data quality Satisfaction with content and extensiveness of reminders Attitudes toward the system
Acceptability: the degree to which the user perceives the PC CDS technology is appropriate, adequate, and relevant and that both clinicians and patients see the CDS^b^ as helpful [58].	Measuring acceptability should involve attention to user subgroups who have different preferences and needs regarding guidelines and alerts.	Sustained use of the PC CDS technology Perceived benefit for patient case and complexity User perceptions of impact on performance
Patient health outcomes: the degree to which change in health status is attributable to PC CDS interventions [21,23,36,45,46,57,59].	Measuring patient health outcomes involves examining changes in health status relevant to the disease or condition attributable to PC CDS [23]. As with many other subdomains, measures should also consider the outcomes patients desire in addition to traditional clinical indicators.	Life expectancy Quality of life Morbidity Mortality Burden of disease Changes in PRO^c^ scores due to intervention Changes in patients’ self-reported symptoms and symptom burden
Usability: the degree to which the system enables users to carry out their tasks safely, effectively, efficiently, and enjoyably [23].	Usability assessments need to account for the different methods and settings in which the PC CDS technology will be delivered to patients and clinicians [18,21,36,38,46,57,60-65]. Important concepts within usability include how the PC CDS fits with clinicians’ workflows and patients’ lifestyles, learnability of the PC CDS, and the degree to which the PC CDS is used [21,23,36,38,46,59].	Perceived ease of use Rate of usability errors Extent of feature use Help-desk requests Rate of uptake Extent of uptake Persistence of use of alternatives and work-arounds Perceptions of alignment with patients’ daily lives
Clinician performance: the degree to which the clinician improves diagnosis or provides more complete preventive care, better disease management, more accurate drug dosing, and drug prescribing [66].	Evaluators should assess whether technology fosters greater rates of patient-centered decisions among clinicians.	Changes in clinical practice Alert follow-up actions taken by care teams Clinicians’ medication prescribing patterns Workflow efficiency Degree of shared decision-making
Transparency: the degree to which descriptions of the guideline source, updates to the guidelines (if applicable), and the artifact authors are clear; and the degree to which relevant metadata such as potential conflicts of interest and disclosures related to its development and use are available [67].	Information needs to be detailed enough for general understanding of the subject and available in a clear and readable manner to promote patient trust in the system and empowerment to make better-informed decisions [68].	Updates to the guidelines Artifact author Potential conflicts of interest Sources of information provided Feedback on success and error or failure cases

^a^PC CDS: patient-centered clinical decision support.

^b^CDS: clinical decision support.

^c^PRO: patient-reported outcome.

#### Efficient

PC CDS technology should support the intended goal of the intervention while enabling clinicians and patients to complete tasks without wasted or unnecessary effort. PC CDS technology holds promises to increase the efficiency of the clinician effort and other resources needed to care for patients. However, PC CDS technology can increase workload and burden when not designed or implemented properly [51]. PC CDS that is disease- or use case–specific can minimize efficiency and applicability across systems. Previous studies have shown that many CDS systems target a single chronic condition [6], which is a critical gap given that 27.2% of US adults have multiple chronic conditions [69]. This results in CDS that is less scalable and less frequently used, particularly in primary care settings where clinicians do not have time to sort through multiple unrelated and often contradictory PC CDS alerts during a single patient encounter [70]. PC CDS that incorporates patient-specific data based on medical histories or patient-reported outcomes can often facilitate efficiency in prioritizing and integrating recommended medications, referrals, or procedures [70]. In addition, these interventions and user acceptance of them should be monitored to ensure they do not contribute to clinician burnout and are an efficient use of organizational resources. Similarly, PC CDS technology must integrate seamlessly into patients’ lifestyles without increasing the burden on patients or their caregivers.

Efficiency of PC CDS interventions can be assessed by measuring six subdomains: (1) relevance and appropriateness, (2) interoperability, (3) cost, (4) reuse and scalability, (5) service use, and (6) cognitive workload (Table 4).

**Table 4:** Efficient subdomain definitions.

Subdomain definition	Application to PC CDS^a^	Example measures
Relevance and appropriateness: the degree to which recommendations are relevant for the clinical context [21,23,38] and appropriate for patient care [24].	Assessments should take a more holistic view and account for patient outcomes, preferences, and costs versus focusing solely on clinical relevance [71].	Alert appropriateness Prescription appropriateness Appropriateness of treatment Patient perceptions of appropriateness
Interoperability: the degree to which ≥2 systems or elements are able to exchange information and use the information that has been exchanged [18,21,64,72].	Measurement should ensure patient data such as PGHD^b^ can be seamlessly shared between patients and clinicians and between the device and EHR^c^ using data exchange standards [73]. Interoperability metrics can also measure how quickly PC CDS technology can access FHIR^d^ APIs^e^ within EHRs.	Time to write data from patient app to EHR Time to view data from EHR using an app
Cost: the amount of money required to design, develop, implement, and use the system and the amount paid recurrently to use the system [18,23,24,46].	Evaluators should determine their own thresholds for whether interventions perform *well* based on their organizational context [74]. A patient-centered perspective would also examine benefits that are most important to patients and calculate costs to the patient, not only clinicians and health care delivery systems.	Development cost Implementation cost Maintenance cost Hardware and software cost Personnel cost Cost:benefit ratio Financial burden on patient
Reuse and scalability: the degree to which the deployment of CDS^f^ capabilities is expanded with the centralized management of machine-executable knowledge resources, which are then leveraged across multiple care settings by CDS engines interfaced with different health information systems [7,75].	Developing PC CDS artifacts often requires specialized clinical and informatics knowledge that not all health systems have, so it is important to measure how artifacts are being adopted by patients, clinicians, and organizations.	Site uptake of PC CDS technology (number, proportion, or duration of use) Accessibility Organizational perspectives on sustainability Degree of decision task automation
Use of services: the degree to which patients interact with the health care system, the types of care they receive, and the timing of that care [76,77].	Measurements should account for the patient’s perspective on accessibility, continuity, and coordination of care [78].	Use by setting (eg, emergency department and inpatient) Duplicate laboratory tests Time until next appointment Test appropriateness
Cognitive workload: the degree to which the demands placed on a person by mental work are balanced with the person’s mental capacity [18,38,63].	PC CDS technology design should carefully consider the complexity of information and how information is presented to both clinicians and patients.	Patient workload Alert fatigue Think time Cognitive overload Desensitization

^a^PC CDS: patient-centered clinical decision support.

^b^PGHD: patient-generated health data.

^c^EHR: electronic health record.

^d^FHIR: Fast Healthcare Interoperability Resources.

^e^API: application programming interface.

^f^CDS: clinical decision support.

#### Equitable

A plethora of research indicates the persistence of variation in US health care based on personal characteristics and SDOH, yet few studies have examined the impact of health IT on addressing gaps [79-81]. We consider two means by which PC CDS technology could make impacts in health care delivery by (1) unintentionally increasing the digital divide, and thereby who benefits from PC CDS technology [82]; and (2) incorporating SDOH data into decision support, thereby ensuring delivery systems are offering guidance to patients and caregivers that accounts for their specific needs. Given this, key informants emphasized the importance of designing and developing PC CDS interventions with addressing health care variation (and the resulting outcomes of this variation) in mind. For example, organizations should take into account differing levels of education and health literacy in designing technologies. In addition, care should be taken not to use biased clinical algorithms (eg, algorithms that *correct* for race but ultimately lead to undertreatment or overtreatment of minority populations) [83,84].

To measure the overall impact of PC CDS technology, we present 6 factors from the SDOH subdomains [85] that can be used to assess relationships to performance in the framework’s other subdomains. These factors are (1) social context, (2) economic context, (3) organizational and personal health literacy, (4) digital health literacy, (5) physical infrastructure, and (6) health care context (Table 5). These areas of measurement apply both at the individual and organizational levels and across phases of the PC CDS life cycle (eg, differences in usability by social context is important to identify in the design and development phase as well as the use phase).

**Table 5:** Equitable subdomain definitions.

Subdomain definition	Application to PC CDS^a^	Example measures
Social context: the degree to which factors that influence a patient’s social and community supports, including demographics and social cohesion [85].	Social context factors can be used to conduct analyses for other subdomains. For example, patient satisfaction with PC CDS technology can be segmented by patient demographics, such as age, race, ethnicity, sex, education status, and disability status, to ensure technology is satisfactory regardless of demographic differences.	Patient satisfaction with PC CDS technology by preferred language Degree to which PC CDS technology incorporates social context
Economic context: the degree to which factors related to financial status, such as employment, income, and poverty, are included [85].	To optimize patient use of PC CDS technology, measurements should consider the digital divide among users (ie, access to computers and the internet) [86-88].	Use of PC CDS technology by household income Degree to which PC CDS technology incorporates employment context
Health literacy (organizational): the degree to which organizations equitably enable individuals to find, understand, and use information and services to inform health-related decisions and actions for themselves and others [89]. Health literacy (personal): the degree to which individuals have the ability to find, understand, and use information and services to inform health-related decisions and actions for themselves and others [89].	Evaluators should examine whether health literacy impacts performance in other subdomains to understand how their PC CDS and organization are performing in terms of accessibility. For example, a PC CDS tool that delivers information such as decision aids should be assessed for differences in uptake by literacy levels.	Patient engagement with PC CDS technology by education level Literacy level of information provided by PC CDS technology Languages and translations offered by PC CDS technology
Digital health literacy: the ability to seek, find, understand, and appraise health information from electronic sources and apply the knowledge gained to address or solve a health problem [90].	Similar to health literacy, evaluators should examine whether digital health literacy impacts performance in other subdomains, such as engagement and use of PC CDS technology.	Digital health literacy level of information provided by PC CDS technology Patient engagement with PC CDS technology by digital health literacy level
Physical infrastructure: the degree to which factors related to the community in which the patient lives (eg, housing, transportation, and food availability) are included [85].	Although accessing and collecting this information is challenging, PC CDS technology that can do so will be able to tailor better interventions to the specific needs and circumstances of patients. For example, frequent recommendations to schedule a clinic appointment may need to account for a patient’s access to transportation and make available other options such as telemedicine.	Patient engagement with PC CDS technology by housing status Degree to which recommendations of PC CDS technology incorporate access to transportation
Health care context: the degree to which availability of, use of, and attitudes toward health care services impact participation in PC CDS interventions and behaviors after the intervention.	Evaluators should include whether the care is high quality, culturally and linguistically appropriate, and health literate; access to insurance; rurality; attitudes toward health care; and service use in their assessment of health care context [85].	Use of PC CDS technology by attitudes toward health care Patient engagement with recommendations of PC CDS technology by health insurance status Degree to which PC CDS technology incorporates health care context

^a^PC CDS: patient-centered clinical decision support.

#### Patient Centered

Patient centeredness involves facilitating active partnerships among all patients, families, patient representatives, and health professionals that are effective within the context of the larger health care delivery system [91]. Successful PC CDS technologies require a concerted effort to create accessible and targeted communication based on patient characteristics (eg, health literacy and demographics), including finding an appropriate balance between providing needed information and overwhelming people with interruptive alerts [91]. It also means extending decision support beyond merely providing information to identifying resources for patients and their caregivers [91]. One key informant noted the following: “CDS isn’t always thought of as an educational intervention, but when it comes to patients it’s worth considering if patients took away a somewhat lasting knowledge about the situation.”

As PC CDS technologies continue to expand, it is crucial that they are effective in helping patients and caregivers make informed choices about their care and engage, as needed, in shared decision-making discussions with their clinicians and care teams. Measures in the patient centered domain should be made from the patients’ perspectives as much as possible.

Patient centeredness of PC CDS technology can be measured with eight subdomains: (1) patient activation, (2) patient engagement, (3) patient satisfaction, (4) shared decision-making, (5) patient decision-making, (6) patient-relevant outcomes, (7) decisional quality, and (8) patient knowledge acquisition (Table 6).

**Table 6:** Patient centered subdomain definitions.

Subdomain definition	Application to PC CDS^a^	Example measures
Patient activation: the degree to which the patient (1) believes their role is important, (2) has the confidence and knowledge necessary to take action, (3) actually takes action to maintain and improve their own health, and (4) persists in their efforts even under stress [23,92].	Patient activation is an indicator that patients are not only using or satisfied with technology but are being activated by it to manage their health. It is important to consider the influence of behavioral determinants, such as a patient’s knowledge, skills, or usual habits related to PC CDS technology and their health.	Belief that their role in health care is important Confidence to maintain lifestyle changes Degree of patients’ implication in making decisions about their lives Patient locus of control
Patient engagement: the degree to which patients, families, their representatives, and health professionals work in active partnership at all levels across the health care system—direct care, organizational design and governance, and policy making—to improve health and health care [93].	Patients and caregivers can be engaged in PC CDS evidence generation, design, development, implementation, use, and health care delivery [6]. Engagement can be measured on a continuum, reflecting both the level of activity and the extent of decision-making authority a patient has [94].	Extent of participation in technology design processes Extent of use of PC CDS technology Degree of behavior change due to technology
Patient satisfaction: the patient’s experience in using the system and the system’s potential impact [18,24,36,46,57].	This is a subset of user satisfaction that focuses on patient preferences and expectations. This can be assessed even if the patient is not the main or only user of the system.	Ease of decision Adherence to the decision made Patient satisfaction with the clinical encounter Patient trust in the PC CDS tool
Shared decision-making: the degree to which (1) a clinician communicates to the patient personalized information about options, outcomes, probabilities, and uncertainties of available options and (2) a patient communicates values and the relative importance of benefits and harms [95].	This can occur through conversation between the patient and clinician or through the PC CDS technology. Assessment relies on access to the latest evidence regarding treatment options, direction on how to weigh pros and cons, and a clinical culture that is supportive of patients [95].	Decisional conflict Decision regret Decision readiness Use and effectiveness of decision aid
Patient decision-making: the degree to which a patient (1) wants to be involved with CDS^b^ and (2) makes choices on test, treatment, or outcome options based on their values, experiences, and assessments of benefits and harms [96-98]. Patient decision-making can occur with or without clinician involvement (ie, shared decision-making).	Measures should be selected based on what matters to the patient, how much it matters, and perceptions of risks and benefits for their lives [99].	How the decision was made Desire to make medical decisions Desire to seek information
Patient-relevant outcomes: the degree to which patients can choose the outcomes that match their desires, beliefs, goals, and circumstances [99,100].	This is particularly salient for outcomes related to quality of life [101].	Symptoms Adverse events and complications Survival and mortality Pain
Decisional quality: the degree to which patients feel satisfied during and after the decision-making process [102,103].	Measurements of decisional quality should be sensitive to the preferences of patients, that is, account for the relative importance a patient attaches to various outcomes [102].	Informed choice Weighing risks and benefits Confidence in decision Trust in information provided
Patient knowledge acquisition: the degree to which the patient gains lasting knowledge about their medical situation due to the PC CDS tool or intervention [104].	This subdomain should measure the extent to which patients gain knowledge about their medical situations by taking part in the decision support activities [105].	Quality of health information provided Change in knowledge about medical condition

^a^PC CDS: patient-centered clinical decision support.

^b^CDS: clinical decision support.

### Application of the PC CDS Performance Measurement Framework

#### Overview

Implementers and evaluators must make a concerted effort to include patient-centered measures in their assessments, ensuring that these measures address a range of patient-focused factors, such as usability, patient digital and health literacy levels, and integration into patients’ daily lives. To contribute to the field given its nascency, we provide 2 illustrative user scenarios for applying patient-centered measures across different phases of the PC CDS life cycle and levels of measurement (Table 7).

To demonstrate the versatility of the framework, the use cases were drawn to highlight different patient populations (ie, postpartum patients and patients aged >75 years), different potential durations of symptoms (ie, acute and long term), different condition acuities, and different types of PC CDS technology (eg, patient facing and clinician facing). Rather than advocating for the universal measurement of all subdomains, the framework supports a targeted evaluation approach, with implementers and evaluators selecting measures that align with the specific goals and context of their project. The use cases provide only a subset of potential measures from each domain, illustrating how different selections may be appropriate depending on the implementation scope and needs.

**Table 7:** Example use cases for applying the patient-centered clinical decision support (PC CDS) performance measurement framework.

Use case scenario	Relevant domain and subdomains to measure	Measurement level
Design and development of an app for managing HDP^a^ for postpartum patients	Safe: system quality to check if the app has glitches or if it meets users’ needs, such as the ability to add notes to explain high or low readings Timely: care timeliness to assess how quickly patients receive care after reporting clinically significant data Effective: usability of the app to ensure it is easy and enjoyable to use Efficient: interoperability to measure the time it takes to build the app and the amount of effort to integrate with the EHR^b^ Efficient: cognitive workload to assess how the patient and care team use and interpret large volumes of patient blood pressure and symptom data Equitable: health literacy to ensure the app uses accessible language and the health system addresses barriers to accessing and using smartphones or blood pressure monitoring devices Patient centered: patient activation to explore if the app provides patients with confidence and skills to manage their hypertension	Individual user: patient and clinician Population: postpartum patients with HDP Organization: the health care system where the app will be used and the company building the app Health IT system: the health care system’s EHR platform
Use of a stroke risk assessment tool (eg, CHA2DS2-VASc [106]) to help clinicians and patients aged >75 years with a-fib^c^ decide whether to initiate anticoagulation therapy	Safe: error quantification to assess whether the tool safely reduced the patient population’s risk of stroke or adverse events from anticoagulants Timely: information provided when the user is making the decision to see if the assessment is taken when the patient is diagnosed with a-fib rather than after treatment has started or after the patient experiences adverse events Effective: patient health outcomes to measure the impact of the decision on the patient population’s overall lifestyle, physical activity levels, number of adverse events, number of strokes, and other outcomes important to patients Efficient: relevance and appropriateness to ensure the recommendations produced by the tool are based on the clinical condition and context of the patient, such as whether they are physically active and at greater risk of falls Equitable: social context to assess how the tool performs for patients aged >75 years by sex category and race and ethnicity Patient centered: shared decision-making to measure the patients’ engagement with their clinician when deciding to take anticoagulants	Individual user: patient and clinician Population: patients aged >75 years with a-fib Organization: the health care system where the tool is implemented and used Health IT system: the clinical notes or EHR platform where the tool is hosted and calculated

^a^HDP: hypertensive disorders of pregnancy.

^b^EHR: electronic health record.

^c^a-fib: atrial fibrillation.

#### Use Case 1

Hypertensive disorders of pregnancy (HDP) can pose serious health complications and increase the risk of maternal mortality [107]. Postpartum patients with HDP will need to monitor their blood pressure and hypertensive symptoms. To assess the design and development of a patient app to manage HDP, an evaluator could assess the usability of the app through user-centered design principles, ensuring the app is easy and intuitive to use. In addition, patient activation could be assessed in the design phase to explore if the app provides patients with the confidence and skills to manage their hypertension or if additional functionalities are needed, such as an education component. Other important subdomains to measure the system quality of the app include care timeliness, cognitive workload of interpreting blood pressure data, and health literacy considerations in the design of the app.

#### Use Case 2

Patients aged >75 years with atrial fibrillation need to weigh the risks and benefits of taking oral anticoagulants. Anticoagulants can prevent blood clots that cause strokes but can also increase the risk of morbidity and mortality from gastrointestinal and intracerebral bleeding, particularly for older adults at greater risk of falls [108]. The CHA2DS2-VASc tool provides estimates of the risk that patients with atrial fibrillation will have a stroke in the absence of treatment [106], and additional CDS tools provide estimates of a patient’s risk of stroke and bleeding on various treatments. These estimates can help patients, and their clinicians decide whether to initiate anticoagulation therapy. To assess the use of this tool after implementation, an evaluator could consider measurements of shared decision-making to assess the patients’ engagement with their clinician when deciding to take anticoagulants and patient health outcomes to measure the impact of the decision on the patient’s lifestyle, physical activity levels, and other outcomes important to the patient. Other important subdomains to consider would be unintended consequences in terms of adverse events, whether information is provided when the user makes the decision, the relevance and appropriateness of the recommendation provided, and the social context of the population using the tool.

## Discussion

### Principal Findings

#### Overview

In this paper, we describe a new measurement framework that can be used by researchers, operational leaders, and patients to understand the performance and impact of PC CDS technology. The framework incorporates constructs from previously developed health IT and CDS evaluation frameworks and organizes them into measure domains and subdomains. We highlight 5 key considerations for this framework and their relevance.

#### Covers the Entire PC CDS Life Cycle

Domains and subdomains described in this paper address the entire PC CDS life cycle—from the generation of knowledge (which includes creating evidence-based guidelines based on patient-centered outcomes research) to the design, development, and implementation of PC CDS [109], and finally to its use (ie, health care delivery). Some outcome-focused subdomains are more relevant to later stages of the life cycle; for example, patient health outcomes and health care use are most relevant during the use phase, after the technology has been implemented and these outcomes can be observed. On the other hand, subdomains related to the performance of the technology (eg, error quantification and system quality) are relevant across phases but are ideally addressed to the extent possible in the design and development phases. Thus, this framework provides the foundation for a more holistic evaluation of PC CDS technology that covers structure, process, and outcome measures.

#### Direct Focus on the Patient

The framework’s domains and subdomains center on patient aims and specifically include patient centeredness as key components of successful PC CDS technology, representing the first framework to account for the use of PC CDS by patients and clinicians. In addition, each subdomain is a patient-centered concept (eg, patient engagement) or has potential measurements that incorporate patient-centered principles (eg, consideration of patient preferences for when decision support is delivered). By doing so, the framework can facilitate the evaluation of the impact of PC CDS technology on patient involvement in their care decisions; patient-clinician communication through shared decision-making; treatment personalization and holistic care approaches; and, ultimately, patient health outcomes [110]. So far, the emergence of PC CDS technology has revealed a gap in the frameworks, models, and methods commonly used for CDS evaluation, particularly in understanding the effect of including patient data in PC CDS technology and the effectiveness of PC CDS technology [6]. For example, patient engagement is critical to PC CDS interventions, yet few studies have focused on the factors that improve and sustain patient engagement or what types of engagement lead to improved clinical or other outcomes [6]. As artificial intelligence–supported decision support grows, patients’ use of these technologies will likely increase [111]. The framework provides a useful starting point for evaluating and measuring patient use of these tools, what is needed to support broader adoption and use, and the eventual impact of incorporating PC CDS in the patient care process.

#### Covers Measurement at Different Levels

Many frameworks also describe different aggregation levels at which performance metrics can, or should, be most appropriately assessed, depending on the goals of the PC CDS intervention, the nature of the domain and subdomain, and the differing perspectives of user groups (eg, clinicians vs patients). The measurement framework introduced here accounts for the impact of the PC CDS technology occurring at the level of the individual user (patient or clinician), user population, organization, and health IT system level. For example, population-level impacts may be important to evaluators trying to understand the effectiveness of PC CDS interventions on the overall health of the community, and individual-level impacts may be important to evaluators attempting to correlate individual-level use and engagement measures with specific clinical outcome measures to identify the optimal method of PC CDS intervention delivery.

#### Encompasses 6 Independent but Related Domains

Our performance measurement framework provides a balanced methodology to assess the extent of use and the quality of PC CDS interventions. Consequently, it provides 6 relatively independent domains in which measurements can be made and leaves the decision to the user on how to synthesize or prioritize the diverse measurements. An evaluator of an intervention does not need to assess every subdomain in the framework. Rather, they should work to assess ≥1 components of each domain most relevant to the evaluator’s particular PC CDS intervention or research focus. In addition, while we have mapped the measurements and subdomains according to their most common applications, the context and goals of an evaluation may require applications to different or multiple subdomains and domains, respectively. Subdomains and measures may also be closely related and influence each other (eg, patient activation and patient engagement). Given that PC CDS technology implementation occurs in many different contexts that may require departure from a standard framework, our intent is for the framework to serve as a foundation for users as they design their own evaluation plans.

#### Requires Additional Research and Development

We propose this framework as a starting point for PC CDS measurement, but further testing for usability and completeness of the framework is needed to inform refinements as appropriate. We recognize that there has been more work in some of the NAM quality domains than in others. Therefore, some domains are better understood and more fully characterized than others as they pertain to PC CDS technology, including the equitable and patient centered domains. Similarly, some phases of the PC CDS life cycle have been more thoroughly explored than others (eg, the CDS phase has more work done on measurement than the knowledge generation phase, which is a newer area for PC CDS measurement). More work is also needed to define and develop measures for our 6 domains (safe, timely, effective, efficient, equitable, and patient centered), as well as newer domains that have been added to NAM’s framework to support a learning health system [112]. This work will need to consider the emerging use of artificial intelligence in the generation of knowledge and patient-specific advice.

### Conclusions

Taken as a whole, the new, unified PC CDS performance measurement framework we present here provides a firm foundation upon which researchers can build as the field of PC CDS matures. Clearly, more work must be done to better understand what is working, whether the specific PC CDS technology is being used as anticipated, and whether the intended outcomes are being achieved. In addition, we anticipate further development in the prioritization of measures for evaluations, as well as in the identification of relevant patient-centered measures, as PC CDS technologies become more widely available. We expect much progress to be made in PC CDS and its evaluation over the next decade, as our understanding of the needs of patients and clinicians evolves and our ability to collect and interpret more data improves.

## Appendix

Multimedia Appendix 1 Information provided to key informants before participating in the interviews.

## Abbreviations

CDS: clinical decision support
EHR: electronic health record
HDP: hypertensive disorders of pregnancy
NAM: National Academy of Medicine
PC CDS: patient-centered clinical decision support
SDOH: social determinants of health

## Data Availability

The datasets generated and analyzed during this study are not publicly available due to possible reidentification but are available from the corresponding author on reasonable request.
