# Supplementary material for: New Performance Measurement Framework for Realizing Patient-Centered Clinical Decision Support: Qualitative Development Study
Source: J Med Internet Res. 2025 Apr 30;27:e68674. doi: 10.2196/68674 (PMC12079060; doi:10.2196/68674)
Supplement: Multimedia Appendix 1 [file jmir_v27i1e68674_app1.docx]

**Project Goals and Purpose**

The Clinical Decision Support Innovation Collaborative is working to develop a performance measurement framework for assessing patient-centered clinical decision support (PC CDS) interventions. By PC CDS we mean clinical decision support (CDS) that 1) collects and uses new, patient-centric data collected directly from patients (e.g., patient-generated health data [PGHD] and patient-reported outcomes [PROs]), 2) supports asynchronous and synchronous data display and collection, 3) supports shared decision-making between patients and clinicians, and/or 4) involves reminders sent directly to patients via text, smartphone apps, or patient portals.

As part of the process of developing the framework, we conducted a targeted literature review of health IT, CDS, and PC CDS measurement and evaluation frameworks. This document summarizes the results of the literature review.

We used the National Academy of Medicine’s (NAM) six domains of quality^^[[1]](#footnote-2)^^ to organize

important indicators of CDS performance. Our high-level PC CDS performance measurement framework is shown below in Exhibit 1.

**Exhibit 1.** High-level preliminary PC CDS Performance Measurement Framework.

| **National Academy of Medicine Domain** | **Subdomains** |
| --- | --- |
| Safe | - Unintended outcomes - Error quantification - System quality - Completeness |
| Timely | - Computer processing time - Information provided is up to date - Availability - Information is provided when the user is making the decision - Care timeliness |
| Effective | - User (patient or clinician) satisfaction - Acceptability - Patient health outcomes - Cost/cost effectiveness - Usability - Use - Clinician performance - System transparency |
| Efficient | - Relevance/appropriateness - Interoperability - Cost/benefit ratio - Scalability/Reuse - Care utilization - Cognitive workload |
| Equitable | - Social context - Economic context - Organizational and personal health literacy - Physical infrastructure - Healthcare context |
| Patient-Centered | - Patient activation - Patient engagement - Patient satisfaction - Shared decision-making - Patient decision-making - Patient-relevant outcomes - Decisional quality - Patient knowledge acquisition |

Exhibit 2 provides brief definitions for each subdomain listed in Exhibit 1. It also lists the levels at which each subdomain can or should be most appropriately assessed (e.g., patient level versus organization level) depending on the goals of the PC CDS intervention, the nature of the domain and subdomain, and/or the differing perspectives of user groups (e.g., clinicians versus patients).

**Exhibit 2. Subdomain definitions.**

| **Subdomain** | **Definition** | **Level(s) of Measure** |
| --- | --- | --- |
| **Safe** | | |
| **Unintended Outcomes** | The degree to which a specific intervention leads to an unanticipated, and often negative, consequence to the patient, clinician, or healthcare organization. | System, User – Patient and Clinician, Population - Patient and Clinician, Organization |
| **Error Quantification** | The degree to which an intervention leads to patient risks and safety-related reportable adverse events. | Organization, System |
| **System Quality** | The degree to which the information and functions provided by the system meet the user’s needs or expectations and give user satisfaction; the degree to which the system is free from deficiencies or defects. | System |
| **Completeness** | The degree to which the system provides all the information required by the user to make the intended decision or to perform the intended behavior. | System |
| **Timely** | | |
| **Computer processing time** | The time required for the computer to complete the work required to gather data, run logic, and generate an intervention. | System |
| **Whether information provided is up to date** | The degree to which the information presented by the system is based on up-to-date input. | System |
| **Availability** | The degree to which a system is able to be used when the user tries to use it. | System |
| **Whether information is provided when the user is making the decision** | The degree to which the information presented by the system is available at the time it is needed. | User – Patient and Clinician, System |
| **Care timeliness** | The system’s capacity to provide care in a timely manner after a need is recognized. | User – Patient |
| **Effective** | | |
| **User Satisfaction** | The overall evaluation of user’s experience in using the system and the system’s potential impact. | User – Patient and Clinician |
| **Acceptability** | User perceptions that PC CDS is appropriate, adequate, and relevant, and that both clinicians and patients see the CDS as helpful | User – Patient and Clinician |
| **Patient Health Outcomes** | Change in health status attributable to PC CDS interventions. | User – Patient and Clinician, Population – Patient and Clinician |
| **Cost/Cost Effectiveness** | The amount of money required to design, develop, implement and use the system and paid recurrently to use the system. | User – Patient, Organization |
| **Usability** | The degree to which the system enables users to carry out their tasks safely, effectively, efficiently, and enjoyably. | User – Patient and Clinician, Population – Patient and Clinician |
| **Use** | Rate and extent of uptake; persistence of use of alternatives /workarounds (as measured from transactional systems, or self-report). | User – Patient and Clinician, Population – Patient and Clinician |
| **Clinician Performance** | The degree to which the clinician improves diagnosis, provides more complete preventive  care, better disease management, more accurate drug dosing, or drug prescribing, for example. | User – Clinician, Population – Clinician, Organization |
| **System Transparency** | The degree to which descriptions of the guideline source, updates to the guidelines (if applicable), and the artifact author(s) are clear, and that relevant metadata are available. | User – Patient and Clinician, System |
| **Efficient** | | |
| **Relevance/ Appropriateness** | The degree to which recommendations are relevant for the clinical context, and appropriateness for patient care. | System |
| **Interoperability** | The ability of two or more systems or elements to exchange information and to use the information that has been exchanged. | System |
| **Cost/Benefit Ratio** | A way to examine both the costs and health outcomes of one or more interventions. | Organization |
| **Reuse/scalability** | An approach to enabling the widespread deployment of CDS capabilities with the centralized management of machine-executable knowledge resources, which are then leveraged across multiple care settings by CDS engines interfaced with different health information systems. | System, Organization |
| **Care utilization** | The degree to which patients interact with the healthcare system, the types of care they receive, and the timing of that care. | User – Patient, Population – Patient, Organization |
| **Cognitive workload** | The balance of the demands placed on a person by mental work and the person’s mental capacity. | User – Patient and Clinician |
| **Equitable** | | |
| **Social Context** | The degree to which factors that influence a patient’s social and community supports, including demographics and social cohesion. | User and Population - Patient |
| **Economic Context** | The degree to which factors related to financial status such as employment, income, and poverty are included. | User and Population - Patient |
| **Organizational and personal health literacy** | Organizational: The degree to which organizations enable all individuals to find, understand, and use information and services to inform health-related decisions and actions for themselves and others.  Personal: The degree to which individuals have the ability to find, understand, and use information and services to inform health-related decisions and actions for themselves and others. | Organization, User and Population - Patient |
| **Physical Infrastructure** | The degree to which factors related to the community in which the patient lives (e.g., housing, transportation and food availability) are included. | User and Population - Patient |
| **Healthcare context** | The degree to which availability of, use of, and attitudes toward healthcare services impact participation in PC CDS interventions and behaviors after the intervention. | Organization, User and Population - Patient |
| **Patient-Centered** | | |
| **Patient Activation** | The degree to which the patient: 1) believes their role is important, 2) has the knowledge, skills, and confidence to take action, 3) actually takes action to maintain and improve their own health, and 4) stays the course even under stress.. | User and Population – Patient |
| **Patient Engagement** | The degree to which patients, families, their representatives, and health professionals work in active partnership at all levels across the healthcare system—direct care, organizational design and governance, and policy making—to improve health and healthcare. | User and Population – Patient |
| **Patient Satisfaction** | The overall evaluation of the patient’s experience in using the system and the system’s potential impact. | User and Population – Patient |
| **Shared decision-making** | The process by which a clinician communicates to the patient personalized information about options, outcomes, probabilities, and uncertainties of available options, and a patient communicates values and the relative importance of benefits and harms. | User – Patient and Clinician |
| **Patient decision-making** | The process by which a patient a) wants to engage or disengage with CDS, and b) makes choices on test, treatment, or outcome options based on their values, experiences, and assessments of benefits and harms. Patient decision-making can occur with or without clinician involvement (i.e., shared decision-making). | User and Population – Patient |
| **Patient-relevant outcomes** | Outcomes that patients choose depending on their desires, beliefs, goals, and circumstances that reflect what matters to patients.. | User and Population – Patient |
| **Decisional Quality** | The extent to which a patient feels satisfied during and after the decision-making process. | User and Population – Patient |
| **Patient Knowledge Acquisition** | Whether the patient came away with lasting knowledge about their medical situation due to the PC CDS tool/intervention. | User and Population – Patient |
|  |  |  |

1. Institute of Medicine (IOM). Crossing the Quality Chasm: A New Health System for the 21st Century. Washington, D.C: National Academy Press; 2001. [↑](#footnote-ref-2)
